# Development of permanent magnet MnAlC/polymer composites and flexible filament for bonding and 3D-printing technologies

**DOI:** 10.1080/14686996.2018.1471321

**Published:** 2018-05-30

**Authors:** Ester M. Palmero, Javier Rial, Javier de Vicente, Julio Camarero, Björn Skårman, Hilmar Vidarsson, Per-Olof Larsson, Alberto Bollero

**Affiliations:** a Division of Permanent Magnets and Applications, IMDEA Nanoscience, Madrid, Spain; b Höganäs AB, Höganäs, Sweden

**Keywords:** Permanent magnet composite, solution casting, polymer, MnAl, filament extrusion, 3D-printing, 50 Energy Materials, 103 Composites, 300 Processing / Synthesis and Recycling, 203 Magnetics / Spintronics / Superconductors

## Abstract

Searching for high-performance permanent magnets components with no limitation in shape and dimensions is highly desired to overcome the present design and manufacturing restrictions, which affect the efficiency of the final devices in energy, automotive and aerospace sectors. Advanced 3D-printing of composite materials and related technologies is an incipient route to achieve functional structures avoiding the limitations of traditional manufacturing. Gas-atomized MnAlC particles combined with polymer have been used in this work for fabricating scalable rare earth-free permanent magnet composites and extruded flexible filaments with continuous length exceeding 10 m. Solution casting has been used to synthesize homogeneous composites with tuned particles content, made of a polyethylene (PE) matrix embedding quasi-spherical particles of the ferromagnetic *τ*-MnAlC phase. A maximum filling factor of 86.5 and 72.3% has been obtained for the composite and the filament after extrusion, respectively. The magnetic measurements reveal no deterioration of the properties of the MnAlC particles after the composite synthesis and filament extrusion. The produced MnAlC/PE materials will serve as precursors for an efficient and scalable design and fabrication of end-products by different processing techniques (polymerized cold-compacted magnets and 3D-printing, respectively) in view of technological applications (from micro electromechanical systems to energy and transport applications).

## Graphical abstract

**Figure:**
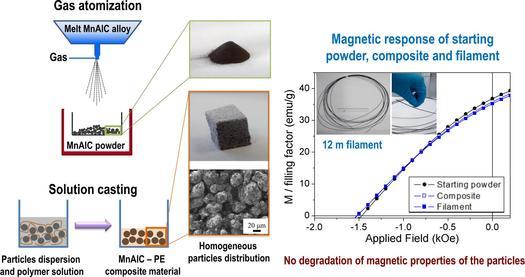

## 1. Introduction

Nowadays, the fabrication of composite materials is attracting much interest due to the possibility of obtaining functional structures by polymerization [1] and advanced 3D-printing [2,3] for applications in novel microelectromechanical systems (MEMS), medicine, electronics, automotive, aeronautics and energy industries [1,4–8]. The composite material is constituted by a polymeric matrix (e.g. polyamide, ABS, polyethylene, polystyrene, among others), which embeds the particles (e.g. magnetic, metallic particles) [9,10]. Specifically, for permanent magnets (PM) applications, the main efforts are focused on the use of NdFeB as the magnetic material in the composite [11,12]. Well-established laser-assisted additive manufacturing used in modern steel industry is not a feasible option for the fabrication of PM components due to the excessively high local temperatures achieved, which will deteriorate irreversibly the PM properties of the starting material. Therefore the necessity to use advanced 3D printing-related technologies to preserve the PM quality during the complete process, from the synthesis of the composite to the filament fabrication and ending with the finally printed magnetic components.

The possibility of fabricating PMs with no shape restriction, attainable by proper 3D-printing technologies, would change the present design and manufacturing model, which is based on magnets only available with pre-defined geometries and little (if any according to machinability of the material) possibility of subsequent shaping. PMs are a crucial element for the development and growth of several high-tech markets such as the energy industry (e.g. a direct-drive wind generator 5 MW contains 3 tons of magnets), the electric automotive industry (e.g. a hybrid car contains 1.4 kg of magnets and a fully electric car is expected to have over 2.5 kg of them), medical applications, and a broad range of electronic products going from home appliances to large industrial equipment. The PMs market moves $6 billion/year and it is increasing with a yearly rate of 7%, forecasting to reach the $25 billion by 2024, with green technologies and electric vehicles counting among the main sectors behind this vertiginous economic growth [13]. Key premises to be fulfilled by improved and/or novel PM materials and related processing technologies to be considered for future industrial implementation are:

(i) Efficient use of supplies while relegating critical raw materials (rare-earths (REs), in particular) to those applications where they are strictly needed.
(ii) Substitution of strategically PMs by feasible alternatives accomplishing:
a. Low cost and abundance of the constituent elements, as many core technological sectors require a large and rapidly increasing volume of these magnets.
b. Lightweight materials since the main market targets demand lighter material to reduce costs (possibility of increasing power in wind turbines, and huge saving in fuel consumption in transport/aerospace applications).
(iii) Design considerations. Efficiently designed end-products (components, sensors, motors…) that dictate PMs shape and size to be implemented, instead of the present technological trend where in-catalogue available magnets (with pre-defined shape and dimensions) are the limiting factors when designing devices.
(iv) Environmental considerations:
a. Environmental friendly ore extraction.
b. Minimum material waste and tooling in the manufacturing process.

MnAl alloy presents advantages as an alternative to magnets based on RE elements for room and low working temperature applications (e.g. sensing, aerospace industry, magnetic resonance imaging, magnetic separation…) [14–16], due to their promising PM properties, provided development of the ferromagnetic *τ*-MnAl phase for enhanced magnetization [14,17]. Recently, coercivity development has been managed in MnAl particles through proper optimization of the phases coexisting in the material and induced microstructural strain [18,19]. The low cost and wide availabilities of Mn and Al in combination with a good machinability, high modulus of elasticity and excellent corrosion resistance complete the characteristics required for making of this material an excellent rare earth-free PM candidate [15,20]. In addition to all these benefits, MnAl shows a reduced density of 5200 kg/m^3^ by comparison with 7600 kg/m^3^ for Nd_2_Fe_14_B, which will lead to lighter PMs.

The synthesis of magnetic composite material faces an important problem in view of practical application as last-generation magnets: a reduced density due to a low filling factor resulting in diluted PM properties [12]. Depending on the physical properties of the magnetic particles and the chosen polymer as holding matrix (i.e. particle size and shape, polymer solubility or melting point), the filling factor of the composite material can be tuned by modifying the amount of magnetic particles in the matrix during the composite synthesis or by varying the fabrication parameters [1]. To authors’ knowledge there is not to date any published study reporting the fabrication of polymer/MnAl-based composites and filament, despite the recent interest for this material as a promising PM candidate.

The industrial implementation of novel PM materials will require the possibility of large-scale production at competitive cost. Gas-atomization is a well-established technique in steel materials production, and it has been recently presented as a promising alternative technique to melt-spinning for the synthesis of PM powder [18,19,21], taking advantage of a high production rate of magnetic powders (tons per hour). The difference between cooling rates (10^5^ K/s for gas-atomization and 10^6^ K/s for melt-spinning) is a disadvantage for preparing slightly over-quenched gas-atomized Nd_2_Fe_14_B powder needed in commercial processes. This is not a limiting factor for the synthesis of MnAl-based powder by gas-atomization [18,19,21], which also provides close-to-spherical shape particles. The latter is an interesting characteristic for the fabrication of polymer/PM-particles composites to be used as source for the fabrication of filament with an enhanced filling factor.

Among the possible composite fabrication techniques, solution casting allows obtaining a homogeneous material in pellets shape, which afterwards can be used as starting material for the fabrication of functional structures [22,23]. Polyethylene (PE) is a polymeric material, which presents an easy solubility to form the composite matrix and suitable physical properties to be combined with MnAlC magnetic powder for room and low temperature technological applications (e.g. aerospace, aeronautics). The relatively low PE melting point (115–135 °C), in comparison with other polymers (e.g. polyamides, polymeric material frequently used for industrial applications, which melting point is over 200 °C [24]) makes it an interesting polymer to be combined with magnetic powders considering a reduced processing temperature, i.e. a lower related cost in view of industrial implementation. Moreover this temperature is sufficiently high to result in PM composites with potential use in a broad range of high-tech applications in energy and transport sectors (sensing devices, actuators, acoustic transducers, fly-wheels…).

Here we report the synthesis process of a composite material formed by MnAlC magnetic particles embedded into a polyethylene polymer matrix with a high filling factor (over 85%) with the aim of fabricate extruded magnetic filament to be used in advanced technologies for PMs fabrication, such as cold-compaction and 3D-printing. The possibility of tailoring the morphology and the magnetic properties of the composite materials and magnetic filament is studied by tuning its filling factor, indicative of the mass of magnetic material into the composites and filaments.

## 2. Experimental section

**Figure 1.**
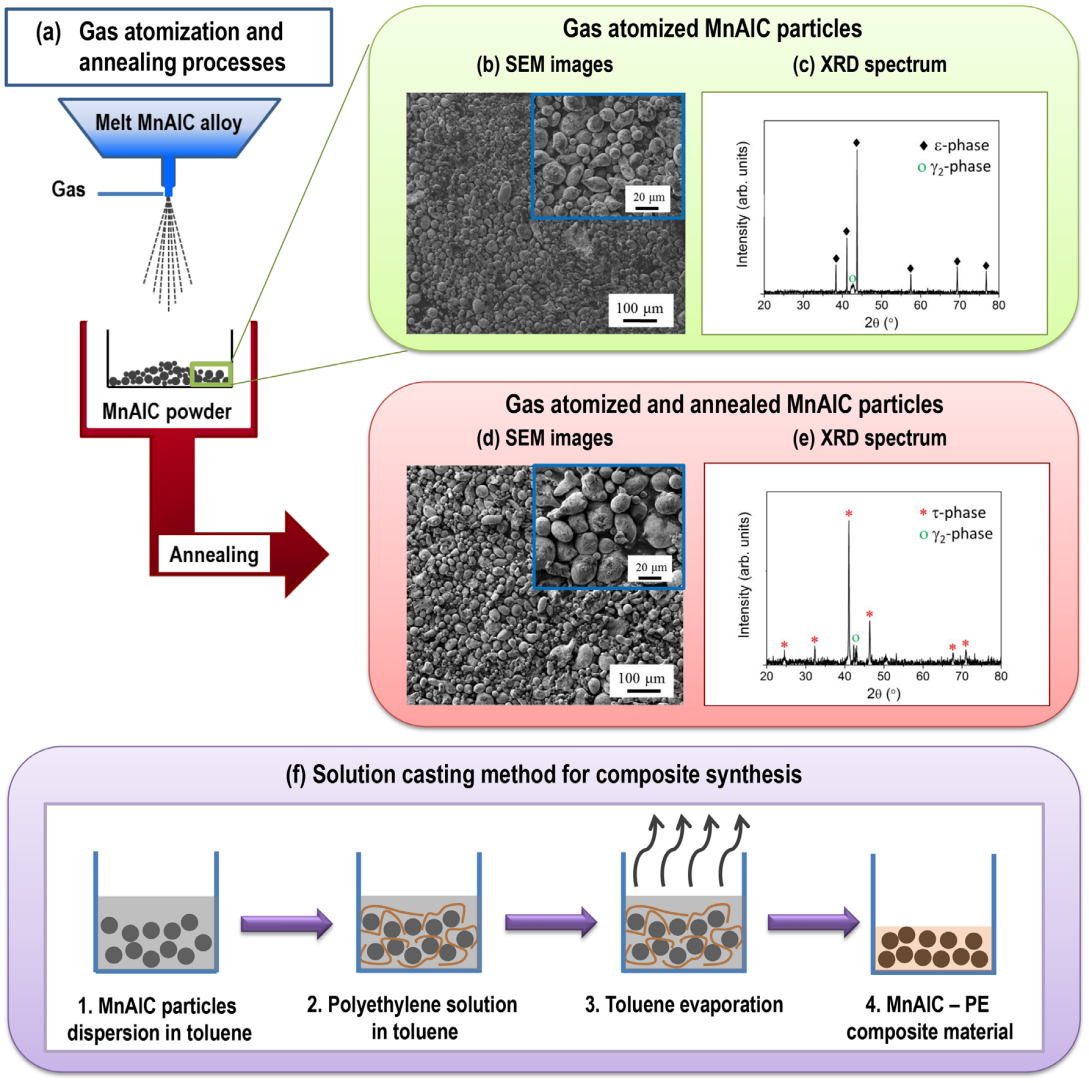
(a) Scheme of the gas atomization and annealing processes of MnAlC alloy to obtain quasi-spherical particles with different crystallographic structure. SEM image showing the microstructure and XRD spectrum of the crystallographic structure of *ε*-phase based particles ((b) and (c), respectively) and *τ*-phase based particles ((d) and (e), respectively). Insets in (b) and (d) show a closer view of the microstructure. (f) Scheme of the solution casting method used for the synthesis of MnAlC – PE composite materials.

Figure 1(a) schematically shows the synthesis of the MnAlC powder by the gas-atomization technique followed by annealing for transformation of the non-ferromagnetic *ε*-phase to the *τ*-MnAlC phase. More details on the synthesis and processing for phases evolution in MnAl-system can be found elsewhere [18,20]. Preparation of the polymer/PM-particles composite is done in a second stage by the solution casting method as illustrated in Figure 1(f) and discussed in the following sections.

### 2.1. MnAlC magnetic powder

The magnetic material used to synthesize the composite was gas-atomized particles which composition was (Mn_57_Al_43_)_100_C_1.19_ (in the following, MnAlC), determined by inductively coupled plasma spectroscopy (ICP). The oxygen content in the alloy was 0.03 wt%, obtained by a LECO Elemental Analyzer. The particle shape was determined by scanning electron microscopy (SEM; Zeiss EVO HD159). SEM image in Figure 1(b) shows that the particles were quasi-spherical with an average diameter below 36 μm.

The crystallographic structure was determined by X-ray diffraction (XRD) using an X’Pert PRO Theta/2Theta diffractometer from Panalytical with Cu-K_α1_ radiation (*λ* = 0.1541 nm). Figure 1(c) shows the XRD pattern of MnAlC particles revealing an *ε*-phase based crystallographic structure. A minor content of *γ*_2_-phase is observed in the pattern as previously reported in other studies [18,20].

Annealing of the as-atomized powder was carried out at 650 °C in nitrogen atmosphere to allow for successful *ε*-to-*τ* phase transformation (Figure 1(c) and (e), respectively). Comparison of SEM images shows that there is no significant microstructural difference after annealing of the as-atomized particles (Figure 1(b) and (d)).

### 2.2. Polymer solution

**Figure 2.**
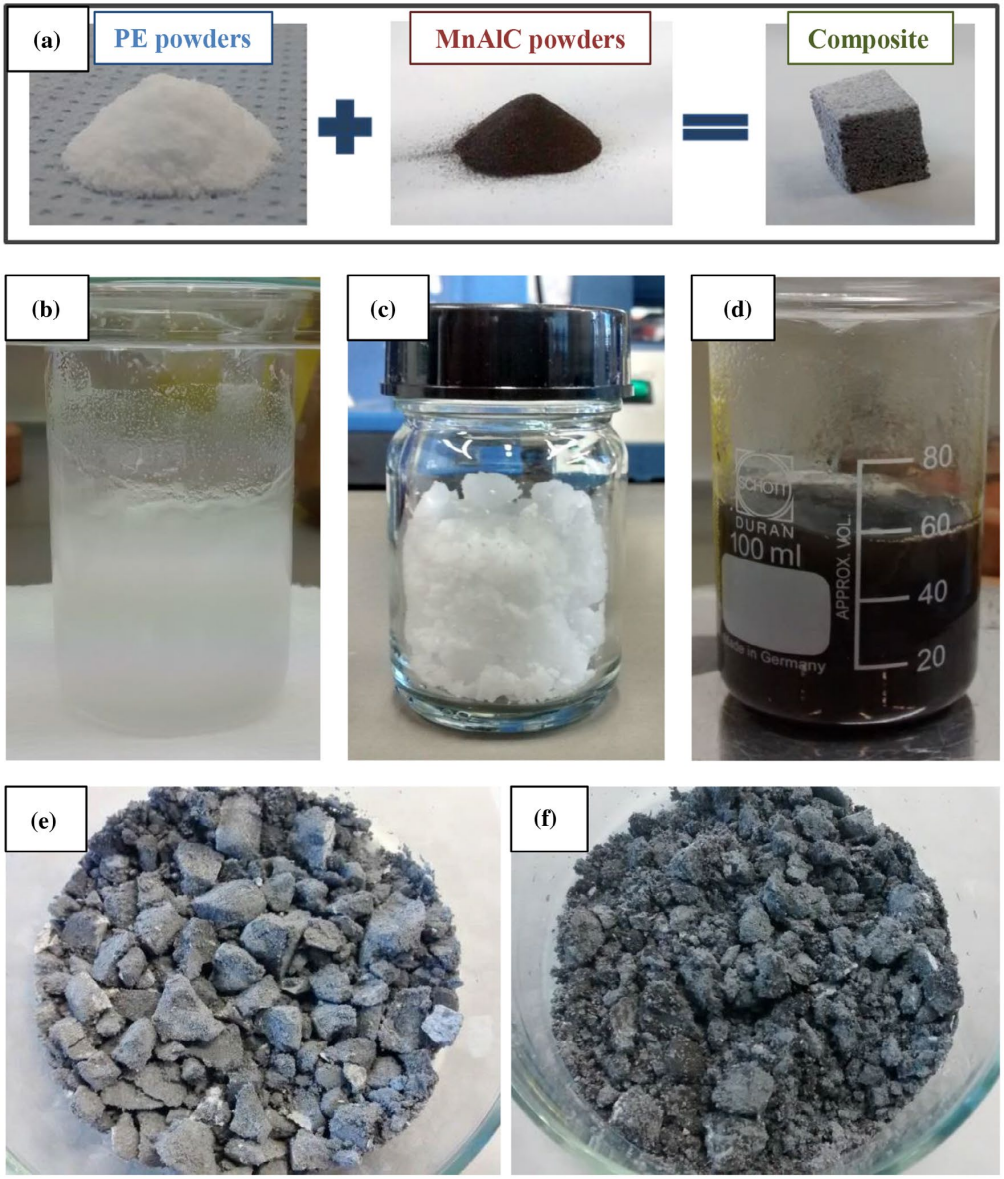
(a) Starting polyethylene (PE), MnAlC powder, and synthesized MnAlC-PE composite; dissolved PE before (b) and after (c) toluene evaporation; (d) PE and MnAlC powders in toluene solution; MnAlC-PE composite with different MnAlC particles content into the PE matrix: (e) 63.1%, and (f) 86.5%.

Composite materials made of MnAlC particles embedded into a PE, (C_2_H_4_)_n_, matrix were synthesized by solution casting method [22,25], being both starting materials in powder-state (Figure 2(a)). Figure 1(f) schematically shows the process. The amount of MnAlC particles into the PE matrix, or filling factor, was tuned by tailoring the mass of materials used during the synthesis process.

In order to determine the proper conditions for the synthesis of the polymeric matrix of the composite material, firstly, a solution of PE powder (6.7% w/w) in 50 mL of toluene was prepared [25,26]. PE was completely dissolved after 1 h in toluene at 85 °C using magnetic stirring (Figure 2(b)). Afterwards, the solution was cooled down to room temperature and the residual toluene was completely evaporated (Figure 2(c)).

### 2.3. Synthesis of magnetic composites

The magnetic composite was synthesized in three steps. Firstly, MnAlC particles were dispersed in 50 mL of toluene using magnetic stirring. While particles dispersion, the solution of magnetic particles and the solvent was heated up from room temperature up to 85 °C, temperature at which the polymer can be solved as determined previously. The amount of MnAlC particles ranged from 4.5 to 7.5 g in order to tune the filling factor of the composite. The amount of magnetic particles in comparison to PE is higher due to the aim of obtaining a composite material with an enhanced filling factor (i.e. over 60%). Secondly, once the temperature was reached and stable, 1.5 g of PE powders were added to the solution (see Figure 2(d)). The solution was kept at 85 °C for 1 h to dissolve the PE and to form a polymeric matrix around the MnAlC particles. Then, the solution temperature was reduced down to room temperature and the solution was kept at that temperature for residual toluene evaporation.

### 2.4. Magnetic filament extrusion

In order to have enough material for filament extrusion, the amount of composite materials was scaled up for each composition. The final composite mass used for extrusion experiments was 25 g. The composites in pellets-like shape shown in Figure 2(e) and (f) were extruded into magnetic filament with cylindrical section using an extruder from Noztek Ltd., based on a screw, which transports the composite from the feeding hopper down to the extrusion nozzle. The filaments were fabricated by applying an extrusion temperature of 120 °C and using a nozzle with a nominal output diameter of 1.75 mm, being both parameters suitable to obtain an extrusion speed of approximately 20 cm/min.

### 2.5. Magnetic characterization

The magnetic properties of the MnAlC powder, composites (MnAlC - PE) and extruded filaments were obtained by measuring the hysteresis loops at room temperature using a vibrating sample magnetometer (VSM) under a maximum applied field of 20 kOe. For the magnetic characterization, five samples were taken from each synthesized composite, and one sample every meter of extruded filament. The values of coercive field (*H*_*c*_), remanence (*M*_*r*_) and magnetization measured at a maximum applied magnetic field of 20 kOe (*M*_20kOe_) were obtained from the hysteresis loops.

## 3. Results and discussion

### 3.1. MnAlC particles embedded into polyethylene matrix

**Figure 3.**
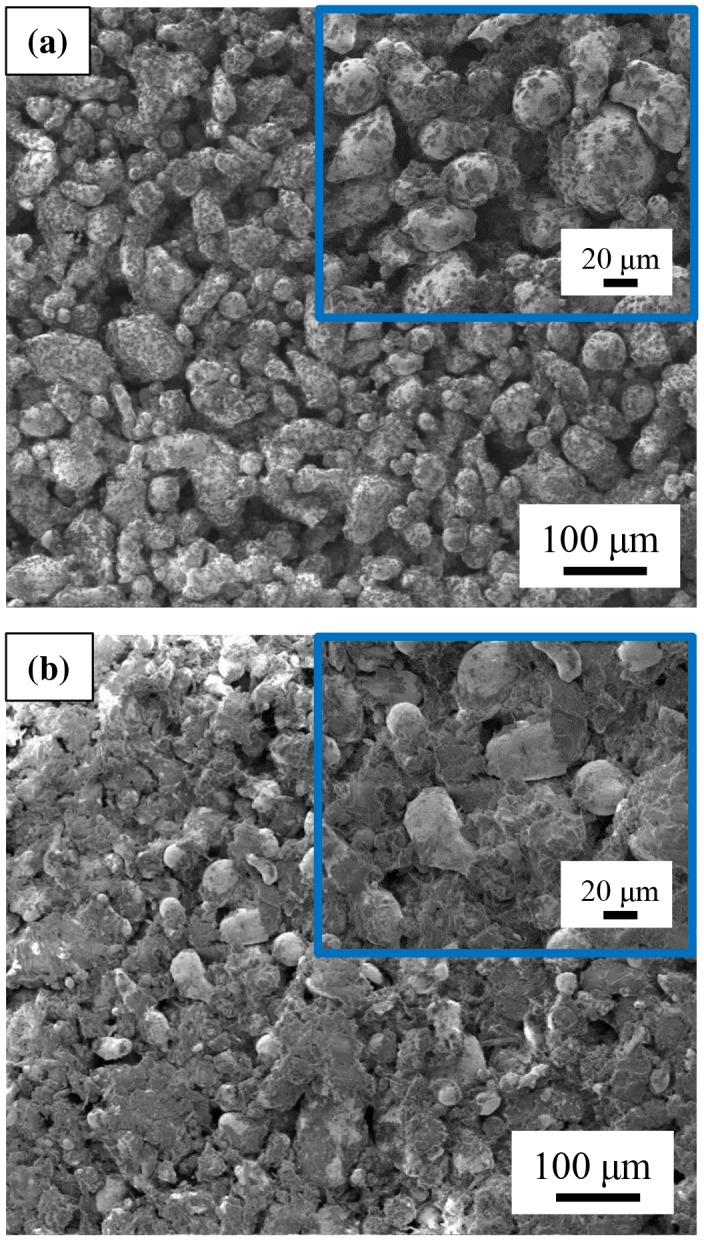
SEM images of composite materials with a filling factor of (a) 86.5% and (b) 63.1%. Insets show detail of the microstructure.

Composite materials with different magnetic particles filling factor were prepared (Figure 2(e) and (f)). SEM images of the composites with a filling factor of 86.5 and 63.1% are shown in Figure 3(a) and (b), respectively. For the composite with MnAlC particles content of 86.5%, PE partially covers the magnetic particles (see dark contrast areas around MnAlC particles in Figure 3(a)). Decreasing the filling factor, PE agglomerates along the composite material. This fact is more evident when the filling factor is lower, i.e. the amount of PE in the composite is higher (see the increased size of the dark contrast areas around magnetic particles in Figure 3(b) for a filling factor of 63.1%).

**Figure 4.**
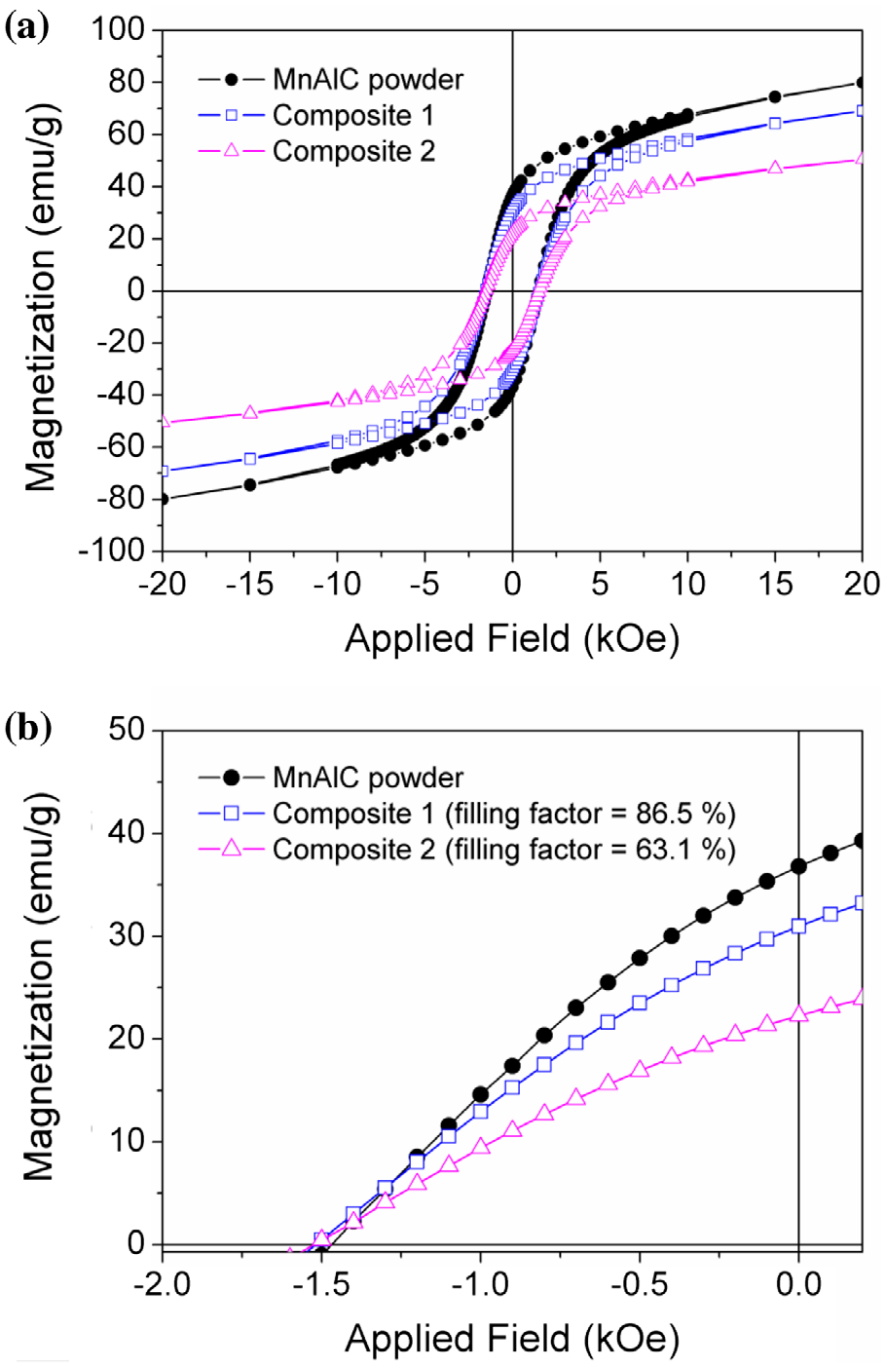
(a) VSM hysteresis loops measured for the starting MnAlC powder and the composites (1 and 2) with different filling factor (86.5 and 63.1% in mass, respectively); and (b) detail of the second quadrant of the hysteresis loops.

The hysteresis loops of MnAlC powder and MnAlC – PE composite materials measured by VSM are plotted in Figure 4(a). Figure 4(b) shows a zoom in of the second quadrant of the hysteresis loops, which allows better observing the difference in the coercive field and remanence values of the samples. The values of the magnetic properties for each material (*H*_*c*_, *M*_*r*_ and *M*_20kOe_) are listed in Table 1. The filling factor of the composites, referred to the mass of magnetic material contained inside, was obtained considering the values of the magnetization at the maximum applied field (*M*_20kOe_) and calculated by the ratio between *M*_20kOe_ for each material and that one for MnAlC powder.

**Table 1.** Magnetic properties of MnAlC powder, MnAlC – PE composite materials and magnetic filaments: coercive field (*H*_*c*_), remanence (*M*_*r*_), and magnetization measured at a maximum applied magnetic field of 20 kOe (*M*_20kOe_).

Sample	Filling factor (%)	*H*_*c*_ (kOe)	*M*_*r*_ (emu/g)	*M*_20kOe_ (emu/g)	*M*_20kOe_*/*filling factor (emu/g)	*M*_*r*_*/*filling factor (emu/g)
MnAlC powder	–	1.47	36.7	80.0	–	–
Composite 1	86.5	1.53	30.6	69.2	80.00	35.38
Composite 2	63.1	1.52	22.2	50.5	80.03	35.18
Filament 1	72.3	1.53	25.5	57.8	79.94	35.27
Filament 2	52.1	1.52	18.4	41.7	80.04	35.32

Note: Values of *M*_20kOe_ and *M*_*r*_ normalized to the corresponding filling factor are included.

The highest values of *M*_*r*_ and *M*_20kOe_ are obtained for MnAlC powder, and they decrease proportionally to the amount of magnetic particles embedded into the polymeric matrix.

Coercivity remains practically unchanged among the different samples and the slight differences (below 4%) may be ascribed to heating effects [18,19] during the composite synthesis. According to the magnetic measurements, a relative standard deviation (RSD) of 7.54 and 7.07% was calculated for the magnetization of the composites 1 and 2, respectively. It is important to remark that in Table 1, the values of *M*_20kOe_ and *M*_*r*_ normalized to the corresponding filling factors show no significant variations for all processed materials (composites and filaments) under study.

**Figure 5.**
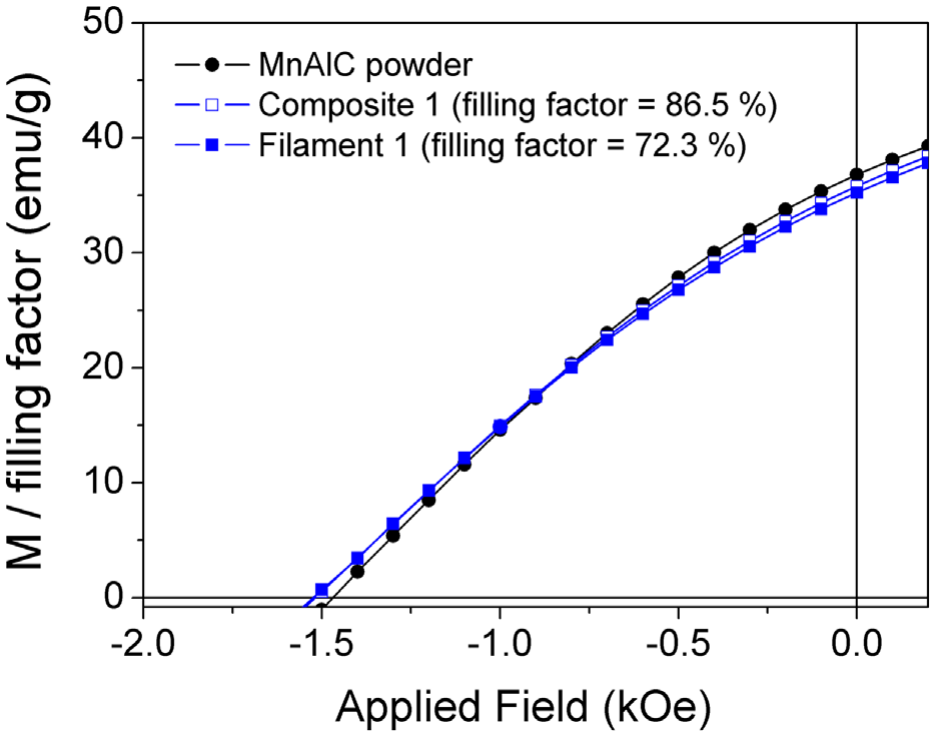
Second quadrant of the hysteresis loops with the magnetization (M) normalized to the filling factors of the composite 1 (86.5%) and the filament 1 (72.3%). Measurement for the starting MnAlC powder is included for comparison.

Figure 5 shows as an example the second quadrant of the hysteresis loop with the magnetization normalized to the filling factor of composite 1 (filling factor of 86.5%). This proves that magnetization values of the starting PM powder are not altered by the chemical products, process and heating used in the composite preparation.

### 3.2. MnAlC magnetic filament

**Figure 6.**
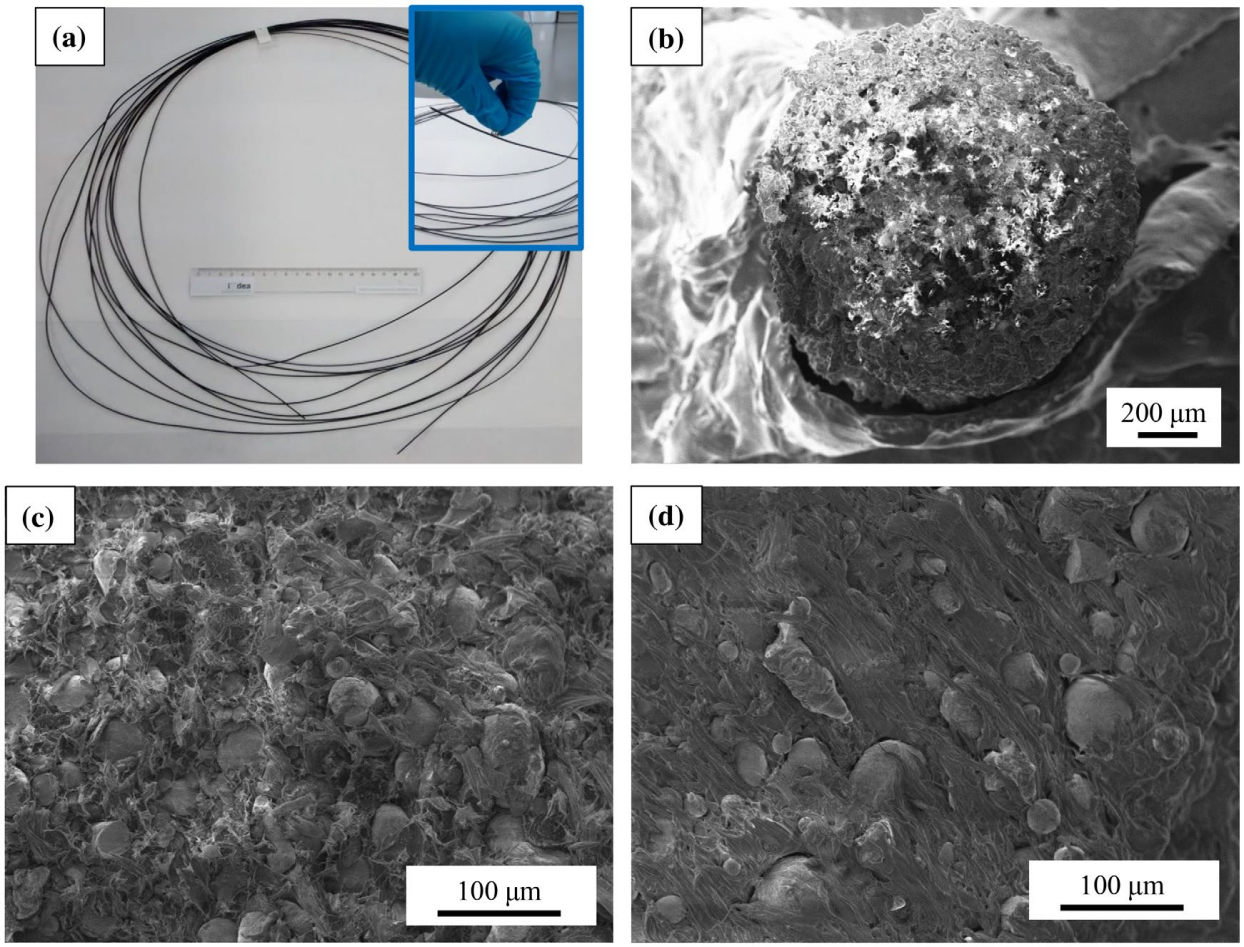
(a) Extruded MnAlC–PE magnetic filament (a 20 cm ruler is included for scale comparison); SEM images of (b) MnAlC–PE filament showing its circular cross section, and internal filament morphology for different filling factors: (c) 72.3% and (d) 52.1%.

Magnetic filaments extruded using the synthetized composite materials were obtained, being to authors’ knowledge the first time that the fabrication of flexible filament of MnAl-based magnetic particles is reported. The composite mass used for the extrusion experiments was enough to obtain 12 m of filament (Figure 6(a)), presenting a magnetic behaviour (inset in Figure 6(a) where the magnetic nature of the filament is evidenced by approaching a commercial PM). The filament shows a homogeneous circular cross section with a diameter of 1.75 mm (Figure 6(b)).

Figure 6(c) and (d) show a closer view of filaments with two different filling factors: 72.3 and 52.1%, respectively. The filaments show a homogeneous morphology, being the MnAlC magnetic particles embedded into the PE matrix, which is identified in the SEM images as the zones with darker contrast.

**Figure 7.**
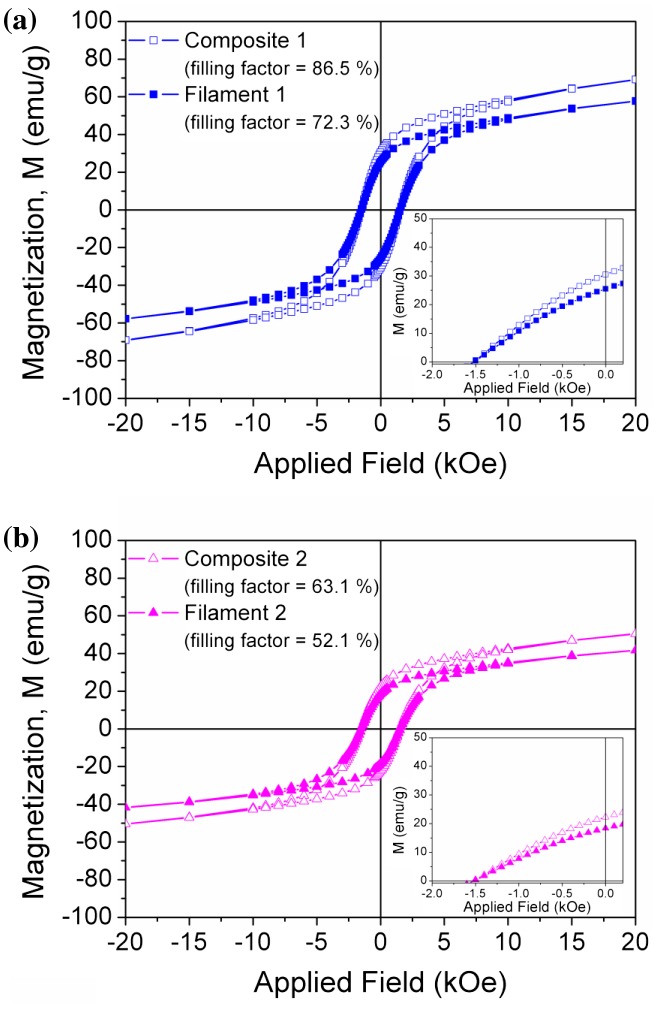
VSM hysteresis loops for extruded MnAlC – PE filaments with different filling factors expressed in mass: (a) 72.3% and (b) 52.1%. Hysteresis loops for the corresponding composites (1 and 2) are included for comparison. In both graphs, a detail of the second quadrant of the hysteresis loops is included as inset.

The hysteresis loops of the magnetic filament measured by VSM are plotted in Figure 7(a) and (b) for the filament with higher and lower magnetic particles content in the polymer matrix, respectively, together with the loops of the corresponding composite material used for each filament extrusion. In both graphs, a zoom in of the second quadrant of the hysteresis loops is plotted to observe the values of *H*_*c*_ and *M*_*r*_. All the magnetic properties determined from the hysteresis loops are listed in Table 1. The magnetic measurements of the extruded filaments show RSD values of 8.05 and 9.88% for the magnetization of the filaments 1 and 2, respectively.

The coercive field for all the magnetic filaments remains almost constant, in accordance to the composite material used for the extrusion. The filaments (labelled as 1 and 2) present lower magnetization values (*M*_*r*_ and *M*_20kOe_) compared to those of the respective composites, which can be explained by the observation that part of the magnetic particles of the composites got stuck in the extruder screw wall during the process due to the exerted pressures, i.e. originating a decrease in the filling factor. The proof that magnetic properties of the starting MnAlC powder are not practically affected by the processing methods (composite fabrication and filament extrusion) comes from the comparison done in Figure 5 and, more in detail, by the values shown in Table 1. Figure 5 shows the second quadrant of the hysteresis loop with the magnetization normalized to the filling factor of filament 1 (filling factor of 72.3%) and the composite 1 (86.5%) used for its preparation are compared together with the magnetic response of the starting *τ*-MnAlC phase powder. It may be observed that hysteresis loops are practically overlapping for the three materials (starting, composite and filament) under study. Moreover, the morphology of the filaments observed by SEM (Figure 6) correlates with the values of the magnetization obtained from the hysteresis loops. Technological work is in progress to manage an increase in the filling factor of the filament, i.e. enhanced magnetization values, by combining technological advances (redesign of the extruder) and material development (combination of MnAlC particles with different sizes and shapes).

## 4. Conclusions

This work correlates the morphological, microstructural and magnetic properties of the first time reported MnAlC magnetic particles-based filament for its use in advanced fabrication techniques such as polymerized compacted magnets and 3D-printing. Homogeneous composites consisting of MnAlC particles embedded in a PE matrix have been prepared through solution casting and used as precursors for filament extrusion. Importantly, in view of practical applications, no deterioration of the PM properties of the MnAlC particles was observed after composite synthesis and filament extrusion, with magnetization values scaling proportionally with the content of magnetic particles in the composite. The coercive field remains almost constant after the fabrication processes with a value of about 1.53 kOe for both the composites and the filaments. In view of potential application of the filament in 3D-printing technologies, a continuous filament with a length of 12 m and a filling factor of 72.3% has been produced in a single batch. The filling factor of the composites and filaments, i.e. the magnetic properties, can be easily tuned during the composite synthesis according to the final application. This study opens a new path for the application of bonding technologies (cold/warm compaction and advanced 3D-printing) to rare earth-free MnAl-based permanent magnet alternatives, allowing for arbitrary designs and realization of more efficient devices.

## Disclosure statement

No potential conflict of interest was reported by the authors.

## Funding

This work was supported by the Spanish Ministerio de Economía y Competitividad (MINECO) through NEXMAG [M-era.Net Programme, Ref. PCIN- 2015-126], ENMA [Ref. MAT2014-56955-R]; 3D-MAGNETOH [Ref. MAT2017-89960-R] projects; Regional Government of Madrid through NANOFRONTMAG project [Ref. S2013/MIT-2850]. JV acknowledges financial support from the Regional Government of Madrid [Ref. PEJ16/IND/TL-1968]. IMDEA Nanociencia is supported by the ‘Severo Ochoa’ Programme for Centres of Excellence in R&D, MINECO [grant number SEV-2016-0686].
